# Observations on Nitrate of Silver Stains of the Conjunctiva

**Published:** 1851-11

**Authors:** James Vose Solomon

**Affiliations:** Surgeon of the Birmingham Eye Infirmary


*Observations on Nitrate of Silver Stains of the Conjunctiva. Case of Absolute Blackness.* By JAMES VOSE SOLOMON, M. R. C. S., Surgeon of the Birmingham Eye Infirmary.—The application of a solution of nitrate of silver, if long and injudiciously applied to the conjunctiva of the eye, produces a discoloration which is indelible. The sclerotic conjunctiva becomes of a dusky brown, or of an olive color; the palpebral linings, more particularly of the lower lid, assume a brownish or livid hue, or, as will be presently shown, become black; the sulcus between the inferior lid and globe is more deeply dyed than the other parts. In the majority of cases the conjunctiva of the superior lid retains its natural color. In a few rare instances the salt becomes incorporated with ulcers of the cornea, forms a subchloride of silver, and perpetuates one or more black lines in their cicatrices.

A still more uncommon, and I believe hitherto unrecorded change of color, consists in absolute blackness of the conjunctiva, an instance of which, the only one that has ever come under my notice, is probably of sufficient interest for publication in your journal.

*Case.*—A young woman, aged 29, came from a small town in Radnorshire to consult me for a dimness of vision.

Both corneæ were extensively covered by opacities, which were irregularly streaked with black lines. The caruncula lachrymalis and tarsal borders were of a jet-black color, giving the appearance, at a cursory glance, of soot or dirt settled on those parts. The palpebral conjunctivæ were smooth, and in color not quite so black as their margins. The sclerotic conjunctiva presented a deep olive color. I found on inquiry, that she had, at one time, suffered from strumous ophthalmia, for the cure of which a strong ointment and some drops had been prescribed and freely used. These applications had been continued for three or four months.

The black streaks which traversed the corneal opacities, and the olived sclerotic conjunctiva, decisively indicated the nature of the other discolorations.

There is a much greater susceptibility to these stains in some individuals than in others. They are more common to adults than children; possibly because ophthalmic disease among the latter is for the most part either of the strumous or purulent kind, in both of which the surface of the eye and its appendages are continually bathed in secretion.

If we excise a portion of discolored sclerotic conjunctiva, a white cicatrix is formed, indicating that the sclerotic maintains its natural color.

At present we are unacquainted with any means for removing the stains under consideration. Our obvious duty is to prevent their occurrence by vigilant attention and care. This may be accomplished by prescribing the preparations of nitrate of silver in short and intermitting courses, and by frequently noting the condition of the lining membrane of the inferior palpebra. In public ophthalmic practice, it would be well if the solution were not dispensed in larger quantities than two drachms at one time.

Since writing the above, a man 60 years of age, who has been all his life a martyr to rheumatism, has become my patient at the Eye Infirmary. The right globe is collapsed; the left eye retains some vision; it has been repeatedly inflamed; the iris is of a dull leaden color, and convex towards the cornea; the pupil is puckered, adherent to the lens, and filled within a third of its area by opaque lymph (artesia irridis imperfecta.) Near the centre of the cornea is a leucoma.

Fifteen or eighteen months ago, he for the first time consulted a surgeon, who prescribed caustic drops, which he has ever since applied. The conjunctiva of the inferior palpebral sinus is of a greenish black color. The inner surface of the superior lid has lost somewhat of its natural polish; a few black drops assume an arborescent shape near the superior punctum; a light brown and well-defined narrow stripe extends along the concave aspect of its tarsal cartilage.

I have cited this case to show the impropriety of allowing patients to use the preparations of lunar caustic *ad libitum;* and as an interesting example of how well the superior lid escapes serious change of color, even in the worst and most neglected cases. It also illustrates the destructive character of uncontrolled rheumatic ophthalmia.—*London Medical Times.*

